# Integrating GWAS-guided markers preselection with genomic selection enhances prediction of pulpwood-related traits in slash pine (*Pinus elliottii* Englem.)

**DOI:** 10.1186/s12870-026-09114-4

**Published:** 2026-05-28

**Authors:** Yadi Wu, Xianyin Ding, Shu Diao, Qinyun Huang, Guiqi Shang, Zifeng Tan, Shaoze Wu, Xiahui Hua, Chengbo He, Qifu Luan, Zhi-Qiang Chen, Harry X. Wu

**Affiliations:** 1 Research Institution of Subtropical Forestry, Chinese Academy of Forestry Key Laboratory of National Forestry and Grassland Administration On Forest Cultivation and Management in East China, Hangzhou, 311400 China; 2 College of Forestry, Northeast Forestry University, Harbin, 150040 China; 3 College of Life Sciences, Henan Normal University, Xinxiang, 453007 China; 4 State Key Laboratory of Tree Genetics and Breeding, Co-Innovation Center for Sustainable Forestry in Southern China, Nanjing Forestry University, Nanjing, 210037 China; 5 Department of Forest Genetics and Plant Physiology, Umeå Plant Science Center, Swedish University of Agricultural Sciences, 90187 Umeå, Sweden

**Keywords:** Slash pine, Genomic selection, SNP preselection, Bayesian Lasso, Pulpwood properties

## Abstract

This study aimed to enhance the efficiency of genomic prediction for pulpwood-related traits in slash pine (*Pinus elliottii* Engelm. var. *elliottii*) by integrating genome-wide association study (GWAS) information with genomic selection (GS). We evaluated 12 traits related to growth, fiber, and wood chemical composition in a population of 340 individuals genotyped with 319,286 high-quality SNPs, comparing the performance of six GS models, including GBLUP and Bayesian methods, under varying training population sizes and marker densities. The results showed that while both GBLUP and Bayesian Lasso performed well, Bayesian Lasso slightly outperformed GBLUP for fiber traits. Predictive ability (PA) plateaued at approximately 100 K SNPs for fiber traits, 60 K for DBH, and 10 K for wood chemical composition traits in all models. Using 100 K random SNPs, PA ranged from 0.05 to 0.23, which expanded to 0.09–0.35 with GWAS-guided SNP preselection (maximum improvement of 16.26%) and further broadened to 0.01–0.38 by incorporating large-effect QTLs (greatest improvement of 23.54%). Overall, integrating GWAS information into GS frameworks significantly improved prediction accuracy as assessed by t-test, offering a cost-effective strategy to accelerate genetic improvement. These findings provide practical guidance for enhancing breeding efficiency in slash pine and other conifer breeding programs.

**Supplementary Information:**

The online version contains supplementary material available at 10.1186/s12870-026-09114-4.

## Introduction

Genomic selection (GS), is an advanced breeding technique that leverages high-throughput DNA genotyping data to predict the genetic value of individuals [1, 2]. Unlike traditional best linear unbiased prediction (BLUP), which relies solely on phenotypic and pedigree information, GS integrates phenotypic data, pedigree information, and genome-wide marker data to develop prediction models based on a training population, enabling the estimation of genomic breeding values (GEBVs) [3]. GEBVs reflect the genetic merit of individuals and allow for early selection without phenotypic data. The core advantages of GS include: (i) enabling the prediction of unphenotyped individuals, (ii) accelerating breeding cycles and enhancing genetic improvement, and (iii) improving the efficiency of selection for traits characterized by limited heritable variation [4]. Applications in the breeding programs of animals [5], crops [6], and forest trees [7] have consistently demonstrated the potential of GS to deliver greater genetic gains than conventional breeding methods. In forest trees, the accumulation of linkage disequilibrium(LD) through selective breeding and the highly polygenic architecture of complex traits, including growth and wood traits, give GS substantial potential for accurate molecular prediction in tree breeding [8, 9].

The performance of GS is typically assessed using predictive metrics, including prediction ability (PA) and prediction accuracy (PC). PA is defined as the Pearson correlation between observed phenotypic values and GEBVs calculated from an independent validation set. Because observed phenotypes contain both genetic and environmental components, PA can be constrained by trait heritability. Therefore, PC is derived by standardizing PA with the square root of the trait’s narrow-sense heritability(*h*^2^) [3, 10]. This heritability adjustment provides a more comparable measure of prediction performance across traits with different heritability levels than reporting PA alone [11–13]. In principle, GS efficiency depends on the model’s capacity to capture quantitative trait locus (QTL) effects through LD [14, 15]. Key factors influencing predictive performance include training population (TP) size, marker number and density, and the statistical model employed. Widely used statistical models include parametric approaches such as GBLUP, BayesA, BayesB, Bayesian LASSO, as well as non-parametric algorithms such as random forest support vector machines [15, 16]. While differences in PA across models are often minor in tree breeding applications [17], they may become critical for complex traits or when marker distribution is uneven [18, 19].

Generally, increasing TP sizes enhances the ability to capture the LD patterns and improves prediction performance. And, studies in forest trees have demonstrated that a few thousand randomly selected SNPs are often sufficient to capture most genetic variance and achieve PA comparable to models using all available markers in cross-validation of the same generation [8, 20]. Moreover, marker preselection strategies can further improve prediction performance in some cases. By prioritizing SNPs strongly associated with QTLs, predictive models can more effectively capture trait-associated genetic variation [8, 21]. Recent studies have shown that incorporating large-effect SNPs identified through GWAS as fixed effects in GS models can substantially improve accuracy, a strategy validated across multiple crop species and simulation studies [8, 20]. Conifers present unique challenges for GS due to their large and complex genomes, often exceeding 20 Gb in size, coupled with intricate LD patterns. Advances in high-throughput genotyping and the reduction in sequencing costs have facilitated the development and application of various genotyping platforms, including SNP arrays, exome capture sequencing, and genotyping-by-sequencing (GBS) [22]. These tools have now been successfully applied in major conifer species such as Norway spurce (*Picea abies*) [23], Scot pine (*Pinus sylvestris*) [24], Loblolly pine (*Pinus taeda*) [25], Slash pine (*Pinus elliottii*) [26]*,* and Masson pine (*Pinus massoniana*) [27]. The growing accessibility of dense marker resources provides favorable conditions for integrating GWAS results into GS pipelines, making GWAS-informed marker preselection and QTL incorporation practical and effective strategies for improving prediction accuracy in forest tree breeding [8].

Slash pine, introduced to China in the 1930 s, has become an economically important pulpwood tree species in China after nearly a century of domestication [28, 29]. Its favorable fiber properties, fast growth, and stable yield made the species a cornerstone of the regional pulp and paper industry [30, 31]. However, the long breeding cycle and the complex inheritance of pulpwood-related traits such as growth, wood fiber characteristics, and chemical composition constrain the progress of genetic improvement. While GS has shown great promise in forest trees, its systematic application to slash pine pulpwood traits remains limited.

In this study, we applied GS to predict 12 pulpwood-related traits in slash pine, including growth traits (e.g., diameter at breast height), wood fiber characteristics (e.g., fiber length, fiber width, curl index, kink index), and chemical components (e.g., cellulose and lignin contents). The main aim of the study were: (i) estimating the additive genetic variance of pulpwood traits using a genomic relationship matrix and compare it with estimates based on traditional pedigree-based models; (ii) assessing GS prediction efficiency under varying numbers of SNPs, training population sizes, and multiple statistical models; and (iii) evaluating the potential of GWAS-informed marker preselection including the use of major-effect QTLs as fixed effects, for enhancing predictive performance. By addressing these objectives, our study advances the understanding of the genetic architecture of pulpwood traits in slash pine and establishes a practical framework for implementing GS in slash pine breeding programs, with direct implications for accelerating genetic improvement.

## Materials and methods

### Plant materials and experimental design

In April 1976, a clonal of *Pinus elliottii* Engelm. var. *elliottii* seed orchard was conducted in Hanghu Town, Yuhang district, Hangzhou City, Zhejiang Province (30°20′ N, 119°50′ E). The orchard comprised 300 elite clones that were initially identified through provenance trials carried out across seven provinces in China, including Guangdong, Fujian, Jiangxi, Jiangsu, Hunan, Hubei, and Zhejiang. These genetically unrelated clones, characterized by superior growth performance, were established by grafting vigorous crown scions from selected elite trees onto two-year-old slash pine rootstocks.

Open-pollinated seeds were collected from the orchard in 1992, and the resulting seedlings were raised in the following year. In 1994, a progeny test plantation was established at 30°27′ N, 119°49′ E. The trial consisted of 33 families arranged according to a randomized complete block design, with six replicates per family and six-tree row plots. Tree spacing was maintained at 2 m within rows and 3 m between rows. The experimental site is situated in a gently undulating landscape with yellow–red soils of moderate fertility and is characterized by a mean annual precipitation of approximately 1,480 mm and an average annual temperature of 17.0 °C [32].

Phenotypic assessments and sample collections were carried out in March 2024 across six blocks of the progeny trial. The collection of plant materials and all field studies were conducted in accordance with relevant institutional, national, and international guidelines and legislation. Permission to access the site and collect samples was granted by the Changle Forest Farm, which co-manages these genetic resources with the Research Institution of Subtropical Forestry, Chinese Academy of Forestry (RISFCAF). The formal identification of the *Pinus elliottii* Engelm. samples was undertaken by Qifu Luan. Voucher specimens of the studied materials have been deposited in the herbarium of the RISFCAF, under voucher numbers RISF-PEE-2024–001 to RISF-PEE-2024–340. Three trees were sampled per plot. While field sampling, individuals showing visible damage were excluded from the analysis. In total, phenotypic data were obtained from 340 individual trees (Table S1). For each sampled tree, one wood core was collected and used for the measurements of basic wood density, fiber properties, and chemical composition.

#### Phenotype data

1. Diameter at breast height (DBH): trunk diameter was measured at 1.3 m above ground level using a caliper.
2. Basic wood density (BWD): A maximum moisture content method was used to measure the basic density of wood [33]:

1 $$\uprho =\frac{1}{\frac{{\mathrm{M}}_{\mathrm{W}}}{{\mathrm{M}}_{\mathrm{d}}}-0.3464}$$

where $\uprho$ denotes basic density of the wood sample (g·cm^−3^), ${\mathrm{M}}_{\mathrm{W}}$ represents the mass of the wood core when saturated with water, and ${\mathrm{M}}_{\mathrm{d}}$ represents the mass of wood core when completely dry.

(3) The fiber properties were analyzed using a fiber quality analyzer [34] (FQA-360, OpTest Equipment Inc., Canada), including fiber length (FL), fiber width (FW), kink index (KI), total kink angle (TKA), curl index (CI), and percentage of fines (PFF). Fines were classified as fibers with lengths shorter than 0.2 mm and were excluded from the calculation of mean fiber properties.

Mixed earlywood and latewood material was collected from the middle section of each wood core. For each original wood-core sample, three thin transverse sections, each with a fresh weight of less than 50 mg, were prepared as three technical replicates and processed separately in individual test tubes. Each treated subsample was boiled in a water bath for approximately 2.5 h until the wood piece settled at the bottom of the tube. After boiling, a delignification solution consisting of glacial acetic acid and hydrogen peroxide at a 1:1 ratio was added, and the samples were boiled for another 2.5 h until white fibrous solids were isolated from the wood. The delignified fibers were thoroughly rinsed with purified water until the pH of the rinse solution reached neutrality. The analyzer was operated using the manufacturer’s default settings, except that the number of fibers analyzed per sample was specified as approximately 5,000. The mean value across the three technical replicates was used as the tree-level phenotype for each fiber trait in subsequent genomic prediction analyses.

The procedures for the measurement and analysis of pulpwood traits are as follows: Mixed earlywood and latewood samples were collected from the middle section of each wood core and sliced into thin, uniform pieces. The slices were placed into test tubes and subjected to boiling in a water bath for approximately 2.5 h, until the wood pieces settled at the bottom of the tubes. After boiling, a delignification solution (a 1:1 mixture of glacial acetic acid and hydrogen peroxide) was added to the test tubes. The samples were then boiled for another 2.5 h until white fibrous solids were isolated from the wood. The delignified fibers were thoroughly rinsed with purified water until the pH of the rinse solution reached neutrality.

(4) The contents of cellulose (CC) and lignin (LC) were determined according to Chinese agricultural industry standard NY/T 3494–2019 [35], while hemicellulose content (HC) was measured based on the Chinese industrial standard GB/T 745–2003 [36]. For each sampled tree, chemical composition measurements were performed on homogenized wood material from the same wood core with three technical replicates. The mean value of the three technical replicates was then used as the tree-level phenotype for subsequent genomic prediction analyses.

#### Genotype data

New fresh needles were sampled from 340 individuals in 2023. Total genomic DNA was extracted using the M5 HiPer Universal DNA Mini Kit (Beijing Juhemei Biotechnology Co., Ltd., Beijing, China). Genotype data were generated using the 51 K liquid-phased probe array for loblolly and slash pine. To ensure data quality, initial filtering of the captured SNP loci was conducted using VCFtools, excluding sites with a minor allele frequency (MAF) below 0.01 and missing rates exceeding 20%. Finally, 319,286 high quality SNPs were left.

#### Variance component and heritability estimates

All phenotypic data were standardized using Z-scores. A pedigree-based best linear unbiased prediction (PBLUP) model and a genomic-based best linear unbiased prediction (GBLUP) models were fitted for each trait as follows:

The formulas of PBLUP and GBLUP models are as follows:

2 $$\mathrm{y}=\mathrm{X}\upbeta +{\mathrm{Z}}_{1}\mathrm{a}+\in$$

where y refers to the vector of phenotypic observations of a single trait; $\upbeta$ refers the vector of fixed effects, including a grand mean and block effects; a is the vector of additive effects, respectively. $\mathrm{X}$, Z_1_ are the incidence matrices for $\upbeta$, a and $\in$ is the residual, respectively. The pedigree-based relationship (A) was estimated using the ASReml-R V4.0. The genomic-based additive relationship (Ga) matrices were constructed based on imputed SNP data using the R package ASReml-R V4.0 [37, 38].

The narrow-sense heritability was estimated as follows:

3 $${\mathrm{h}}^{2}={\upsigma}_{\mathrm{a}}^{2}/{\upsigma}_{\mathrm{p}}^{2}$$

where ${\upsigma}_{\mathrm{a}}^{2}$ is the estimated additive genetic variance; ${\upsigma}_{\mathrm{p}}^{2}$ is the phenotypic variance, which is the sum of all the variances of the random effects.

The relative goodness-of-fit among different models was evaluated using the Akaike Information Criterion (AIC) and Bayesian Information Criterion (BIC) [39].

4 $$\mathrm{A}\mathrm{I}\mathrm{C}=-2\mathrm{log}\mathrm{L}+2\uprho$$

5 $$\mathrm{B}\mathrm{I}\mathrm{C}=-2\mathrm{log}\mathrm{L}+2\mathrm{log}(\mathrm{n})\uprho$$

where $\mathrm{log}\mathrm{L}$ denotes the REML log-likelihood, $\uprho$ represents the number of estimated parameters, *n* is the number of observations. A smaller AIC or BIC value indicates a better quality of fitness.

#### LD analysis and GWAS

**Fig. 1:**
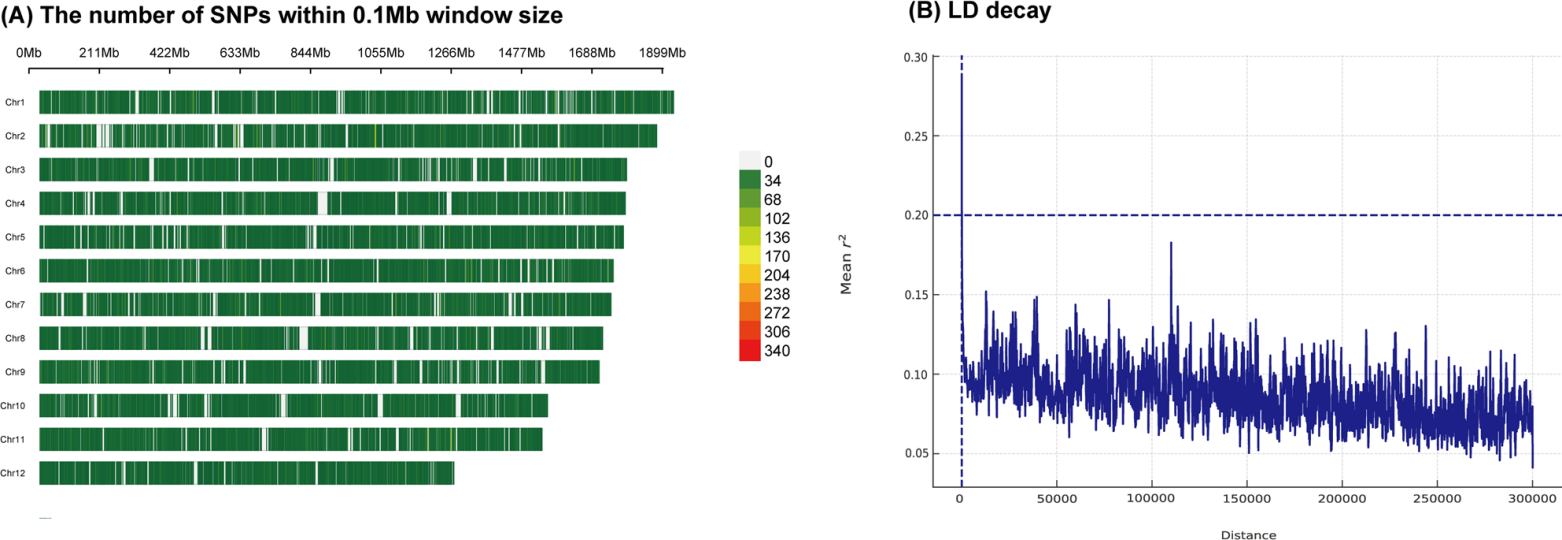
Marker density of SNPs captured by 51 K liquid-phased array and LD decay plot. **A** Marker density of the 51 K liquid-phased array based on 0.1 Mb window size for each of 12 chromosomes. **B** LD decay plot

Linkage disequilibrium (LD) was evaluated at the genome-wide scale using 340 half-sib individuals. All SNP markers were aligned to the slash pine reference genome (unpublished). Among the 319,286 SNPs included on the 51 K liquid-probe array, 317,801 markers were successfully assigned to physical positions and showed a relatively uniform distribution across the 12 chromosomes (Table S2). The remaining 1,485 SNPs could not be reliably anchored to any chromosome and were therefore pooled into an artificial group designated as “chromosome 13”. Pairwise LD statistics (Fig. 1B) within each chromosome were calculated using the PopLDdecay software [40]. PopLDdecay was run with default settings.

**Fig. 2:**
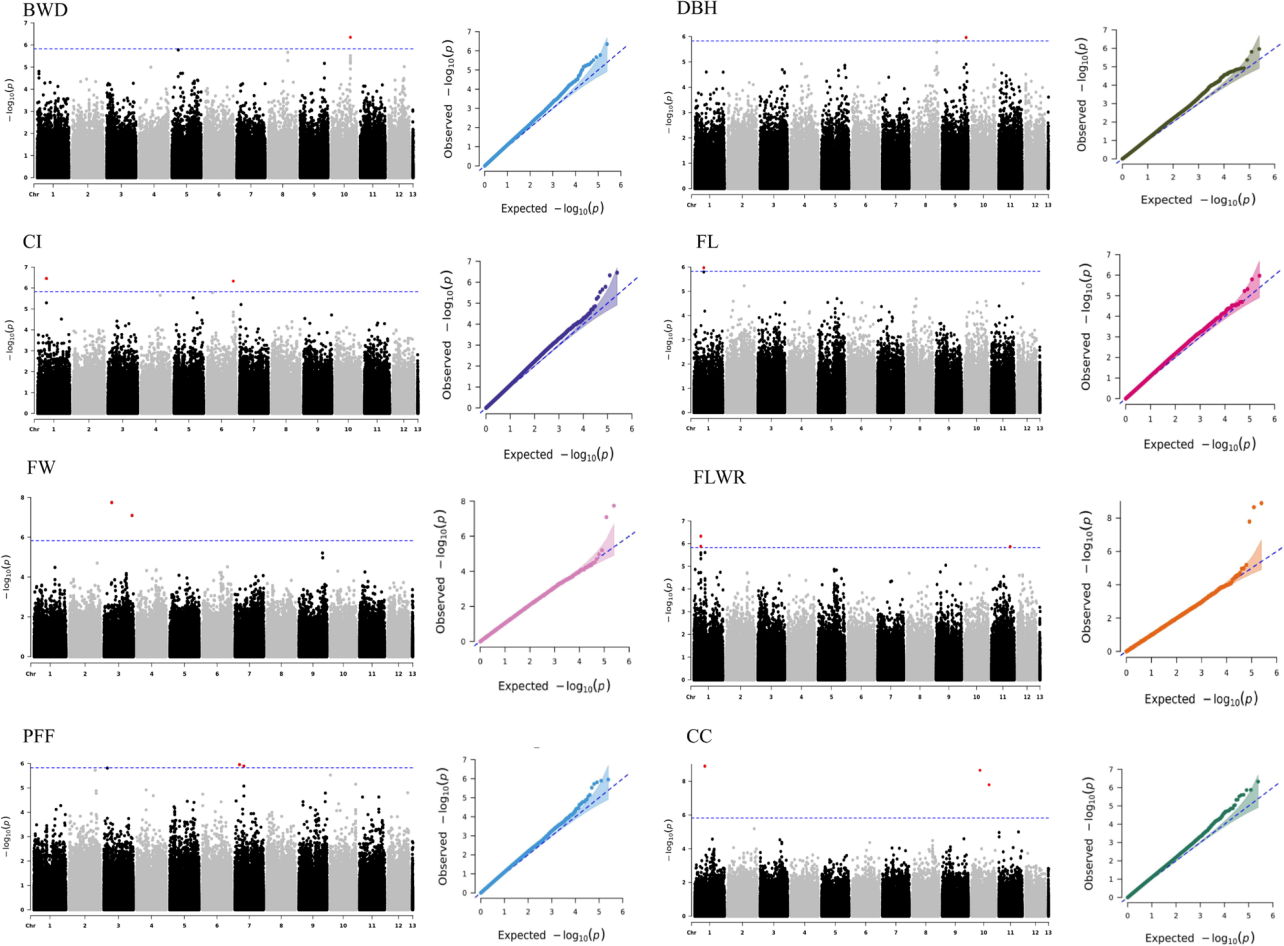
Manhattan and Q-Q plots for eight traits. The blue dashed line represents the significant threshold of *P* = 1.57 × 10^–6^ after the Bonferroni correction. The red dots represent that the SNPs passed the false discovery rate test threshold of 0.05. SNPs not mapped into the Slash pine genome were grouped into a region assumed as chromosome 13 in this study

GWAS analyses were conducted to investigate the additive genetic basis of pulpwood-related traits in the breeding population. Tree-level phenotypic value of each trait was used after z-score standardization. Association mapping was performed on 340 individuals using the BLINK approach implemented in the GAPIT V3.0 software package [41]. To control the effects of population structure, the first three principal components derived from principal component analysis (PCA) were included as covariates in the model. The significance threshold was determined based on the Bonferroni correction, with a cutoff *P-value* of 1.57E-06. Quantile–quantile (Q-Q) plot was used to assess the consistency between observed and expected *P-value* distributions. Marker density (Fig. 1A), Manhattan plots (Fig. 2), and Q-Q plots (Fig. 2) were generated using the R package CMplot.

#### Factors affecting genomic prediction ability

##### Statistical model

To assess genomic prediction performance, six commonly used statistical approaches were compared. Genomic best linear unbiased prediction (GBLUP) was implemented under a mixed linear model framework using the R package ASReml (v4.0). In addition, five Bayesian regression methods, including Bayesian Ridge Regression (BRR), Bayesian LASSO (BL), BayesA, BayesB, and BayesC, were applied to model marker effects. All Bayesian models were fitted using the R package BGLR V1.0.3 [42, 43].

##### Training population size

The influence of training population (TP) size on predictive ability (PA), different proportions of the population were used as training sets, ranging from 10 to 90% in 10% increments. The remaining samples in each case were used as the cross-validation testing set. All 319,286 SNP markers were used to perform seven models. For each trait, pairwise two-sided *t*-tests were used to compare prediction accuracies between SNP selection strategies at the same marker density and between each reduced-SNP model and the model using all available SNPs, with *P* < 0.05 considered statistically significant. For each trait, each preselection strategy was compared with the baseline model with all SNPs using pairwise two-sided paired t-tests, with *P* < 0.05 considered statistically significant.

##### SNPs mark density

To investigate how marker density affects genomic prediction performance, GS analyses were conducted using multiple SNP subsets of increasing size (100, 500, 1 K, 3 K, 6 K, 10 K, 20 K, 40 K、60 K、100 K、200 K and all SNPs). For each trait, each PVE-threshold strategy was compared with the baseline model without fixed-effect SNPs (“None”) using pairwise two-sided paired t-tests, with *P* < 0.05 considered statistically significant.

#### Incorporating SNPs identified by GWAS into genomic prediction

To further evaluate the influence of two different maker preselection strategies on PA value. Firstly, three subsets containing the top 3 K, 50 K, and 100 K SNPs ranked by minimum P values were selected as random effect on the model, and the same number of SNPs was randomly sampled from the entire dataset as controls. Alternatively, to assess the contribution of large effect loci to genomic prediction, we incorporated subsets of SNPs as fixed effects in the GS model according to their proportion of variance explained (PVE). Specifically, SNPs with PVE ≥ 0.0125 were first selected, and then multiple PVE upper thresholds were applied to generate a series of fixed-effect SNP sets (0.0125–0.025, 0.0125–0.050, 0.0125–0.075, 0.0125–0.100, and 0.0125–0.125). For each interval, only SNPs falling within the specified PVE range were included as fixed-effect covariates. BayesLasso models, which showed the highest PA in this study, was used with 90% of the individuals used as the training population for these predictions.

#### Cross-validation test

Apart from analyses focusing on training population size, GS performance was assessed using a repeated tenfold cross-validation framework. For each model, individuals were randomly partitioned such that 90% constituted the training population, while the remaining 10% formed the validation population (VP). This resampling procedure was carried out ten times to improve the stability of the estimates. PA was quantified as the Pearson correlation between genomic estimated breeding values (GEBVs) and the corresponding standardized tree-level phenotypes in the validation set. The prediction accuracy (PC) was derived by standardizing PA with respect to the square root of the trait’s narrow-sense heritability (*h*^2^).

## Results

### Phenotypic variation and quantitative genetic parameters

Descriptive statistics for all measured traits are provided in Table S3. Substantial phenotypic variation was observed among traits (Table 1), indicating broad differences in trait expression within the population. Estimates of narrow-sense heritability (*h*^2^) ranged from 0.07 to 0.81. For fiber traits, heritability estimates were generally moderate, with the highest *h*^2^ observed for FL (0.81) and the lowest for KI (0.18). For the wood chemical component, the heritability of content of lignin (LC) was close to zero under both PBLUP and GBLUP models, whereas moderate heritability were found for cellulose content (CC) and hemicellulose content (HC).

**Table 1:** Additive genetic variance, residual variance, narrow-sense heritability, AIC and BIC values from PBLUP and GBLUP models for twelve phenotypic traits

Traits	Model	${\upsigma}_{\mathrm{a}}^{2}$(SE)	${\upsigma}_{\mathrm{e}}^{2}$(SE)	h^2^	AIC	BIC
DBH DBH	PBLUP	0.23(0.28)	0.98(0.23)	0.19	357.40	368.89
	GBLUP	0.17(0.28)	0.99(0.23)	0.15	272.43	276.04
BWD	PBLUP	0.22(0.35)	0.91(0.27)	0.19	314.70	326.19
BWD	GBLUP	0.3(0.35)	0.91(0.27)	0.25	272.99	276.59
CI	PBLUP	0.67(0.26)	0.56(0.22)	0.54	314.70	326.19
CI	GBLUP	0.85(0.26)	0.54(0.22)	0.61	272.88	276.48
TKA	PBLUP	0.52(0.31)	0.98(0.25)	0.35	314.70	326.19
TKA	GBLUP	0.64(0.31)	0.94(0.25)	0.41	272.96	276.57
KI	PBLUP	0.14(0.11)	0.93(0.14)	0.13	314.70	326.19
KI	GBLUP	0.21(0.11)	0.94(0.14)	0.18	272.97	276.57
FL	PBLUP	0.65(0.26)	0.21(0.22)	0.76	306.58	318.06
FL	GBLUP	0.87(0.26)	0.21(0.22)	0.81	272.93	276.54
FW	PBLUP	0.56(0.18)	0.82(0.17)	0.40	314.70	326.19
FW	GBLUP	0.61(0.18)	0.83(0.17)	0.42	270.59	274.20
FLWR	PBLUP	0.29(0.18)	0.94(0.17)	0.23	337.84	349.33
FLWR	GBLUP	0.31(0.18)	0.91(0.17)	0.26	272.90	276.51
PFF	PBLUP	0.23(0.19)	0.74(0.18)	0.24	345.42	356.91
PFF	GBLUP	0.27(0.19)	0.82(0.18)	0.25	271.87	275.48
CC	PBLUP	0.25(0.2)	0.61(0.19)	0.29	357.40	368.89
CC	GBLUP	0.37(0.2)	0.78(0.19)	0.32	270.86	274.47
HC	PBLUP	0.28(0.28)	0.67(0.23)	0.30	345.53	357.02
HC	GBLUP	0.38(0.28)	0.7(0.23)	0.35	271.38	274.98
LC	PBLUP	0.07(0.17)	0.81(0.17)	0.08	329.79	341.28
LC	GBLUP	0.07(0.17)	0.94(0.17)	0.07	272.88	276.48

(1) Diameter at breast height (DBH), Basic wood density (BWD), fiber length (FL), fiber width (FW), kink index (KI), total kink angle (TKA), curl index (CI), and percentage of fines (PFF), contents of cellulose (CC), Content of lignin (LC), Content of hemicellulose (HC)

(2) ${\upsigma}_{\mathrm{a}}^{2}$(SE) represents Additive genetic variance with its standard error (SE) estimated from the model. ${\upsigma}_{\mathrm{e}}^{2}$(SE) represents Residual variance with standard error (SE). AIC represents akaike information criterion for model comparison. BIC represents bayesian information criterion for model comparison

Comparison of AIC and BIC values across models for the same trait indicated that the GBLUP model provided a superior fit. In general, AIC and BIC values were concordant within the same modeling framework. Estimates of narrow-sense heritability derived from GBLUP were slightly higher than those obtained using PBLUP.

### Summary of 51 K liquid-phased array, LD and association mapping

Using the 51 K liquid-phased SNP array, a total of 319,286 SNPs were captured. A total of 317,801 of all makers were mapped and evenly distributed across the 12 chromosomes (Fig. 1A) in the slash pine genome (unpublished). The number of SNPs per chromosome ranged from 18,487 to 34,721 (Table S2). Based on pairwise LD analysis, the physical distance at which LD decayed to an *r*^2^ value of 0.2 was estimated to be 66.29 kb (Fig. 1B).

GWAS identified 15 SNPs significantly associated with DBH, BWD, CI, FL, FW, FWLR, PFF and CC, under a false discovery rate (FDR) of 0.05 (Fig. 2). The trait-associated SNPs explained 3.16% to 9.66% of the phenotypic variation for wood property traits. No significant SNPs were detected for the remaining wood traits (Table S4).

### Effect of training population size and marker density

**Fig. 3:**
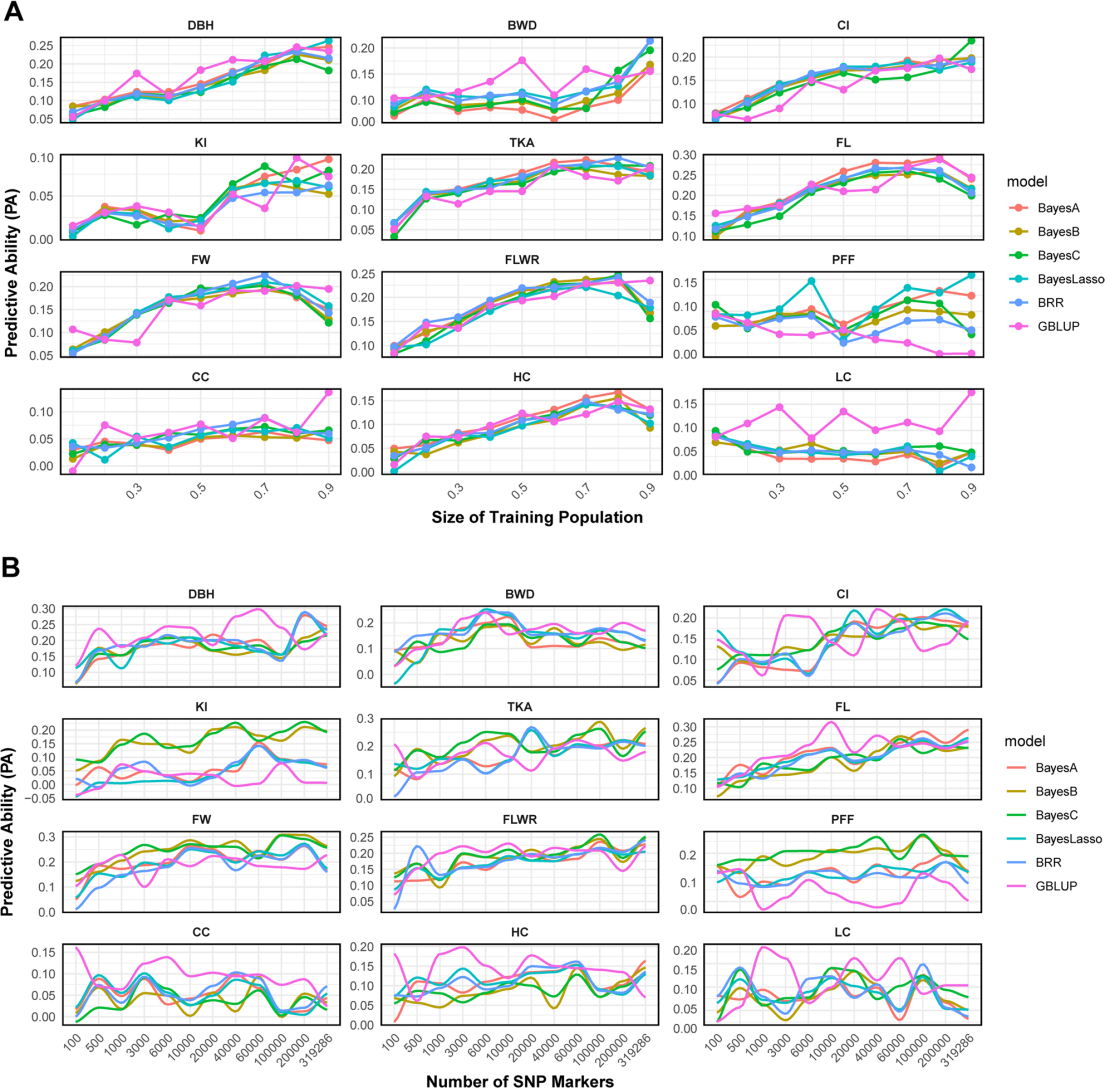
Effect of training population size and marker density on predictive ability (PA) across genomic prediction models. **A** PA across 12 traits under different training population size. **B** PA across 12 traits under a different number of SNPs

All models showed similar increasing patterns of PA as the number of individuals in the TP increased, for all traits (Fig. 3A). In general, the highest PA values were achieved when 70% to 90% of individuals were assigned to TP, suggesting that a larger training set generally enhances prediction accuracy. Notably, traits such as DBH. BWD, CI and FL PA showed a continued upward trend as the TP size increased and did not reach a clear plateau even at 90%, indicating that the current training population has not yet reached saturation for these traits. In contrast, traits such as TKA, FLWR and PFF exhibited more variable responses across TP sizes and models, showing smaller or less consistent improvements when TP exceeded 70%.

The PAs increased with fluctuation for all traits as the number of SNPs increased (Fig. 3B). For DBH, the GBLUP model demonstrated overall superior performance compared to the Bayesian models. However, for most fiber traits, the performance differences between GBLUP and the Bayesian models were not statistically significant. In the case of wood chemical traits, GBLUP outperformed the Bayesian models when the number of markers was within the range of 1,000 to 10,000 (Table S5).

**Fig. 4:**
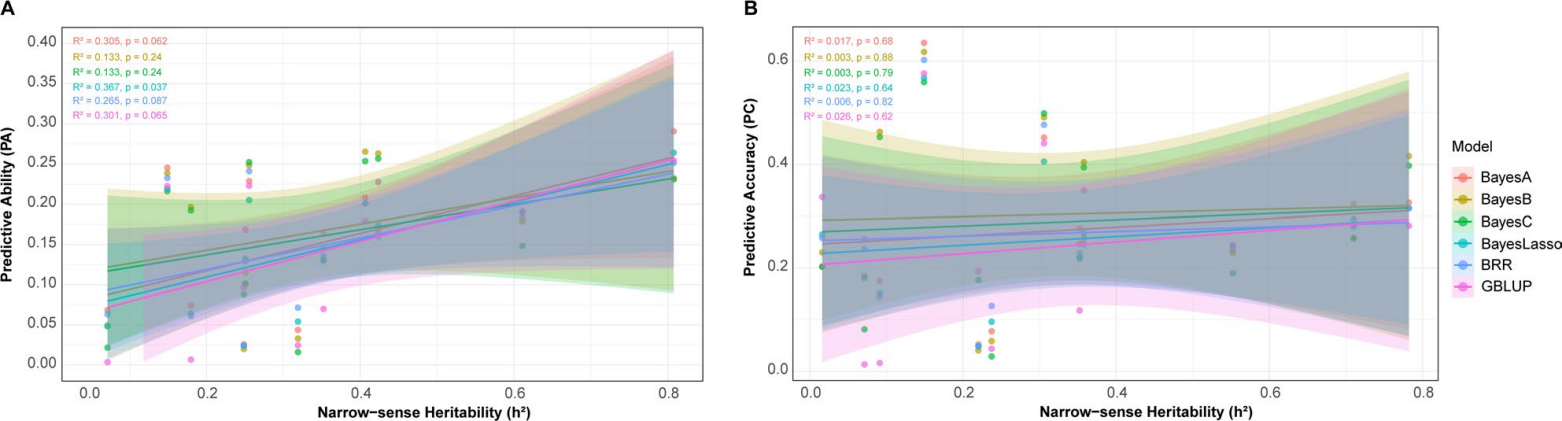
Genomic prediction models and their relationship between trait narrow-sense heritability and both predictive ability and accuracy. **A** Regression between PA and narrow-sense heritability (*h*^2^) across all models. **B** Regression between PC and *h*^2^. Solid lines indicate model-specific trends; shaded areas represent 95% confidence intervals

Among the genomic models, both PA and PC tended to be slightly higher for traits with greater narrow-sense heritability. All models consistently showed a positive, but not significant linear relationship between PA and heritability, furthermore with a negligible relationship between PC and heritability (Fig. 4A and B). Notably, for the BayesLasso model, the coefficient of determination (R^2^) between PA and heritability was the highest across all models, with the smallest associated *P-value*, indicating that the BL model may possess an enhanced capacity to capture genetic variance in traits with higher heritability.

### Integrating association mapping to improve genomic prediction accuracy

**Fig. 5:**
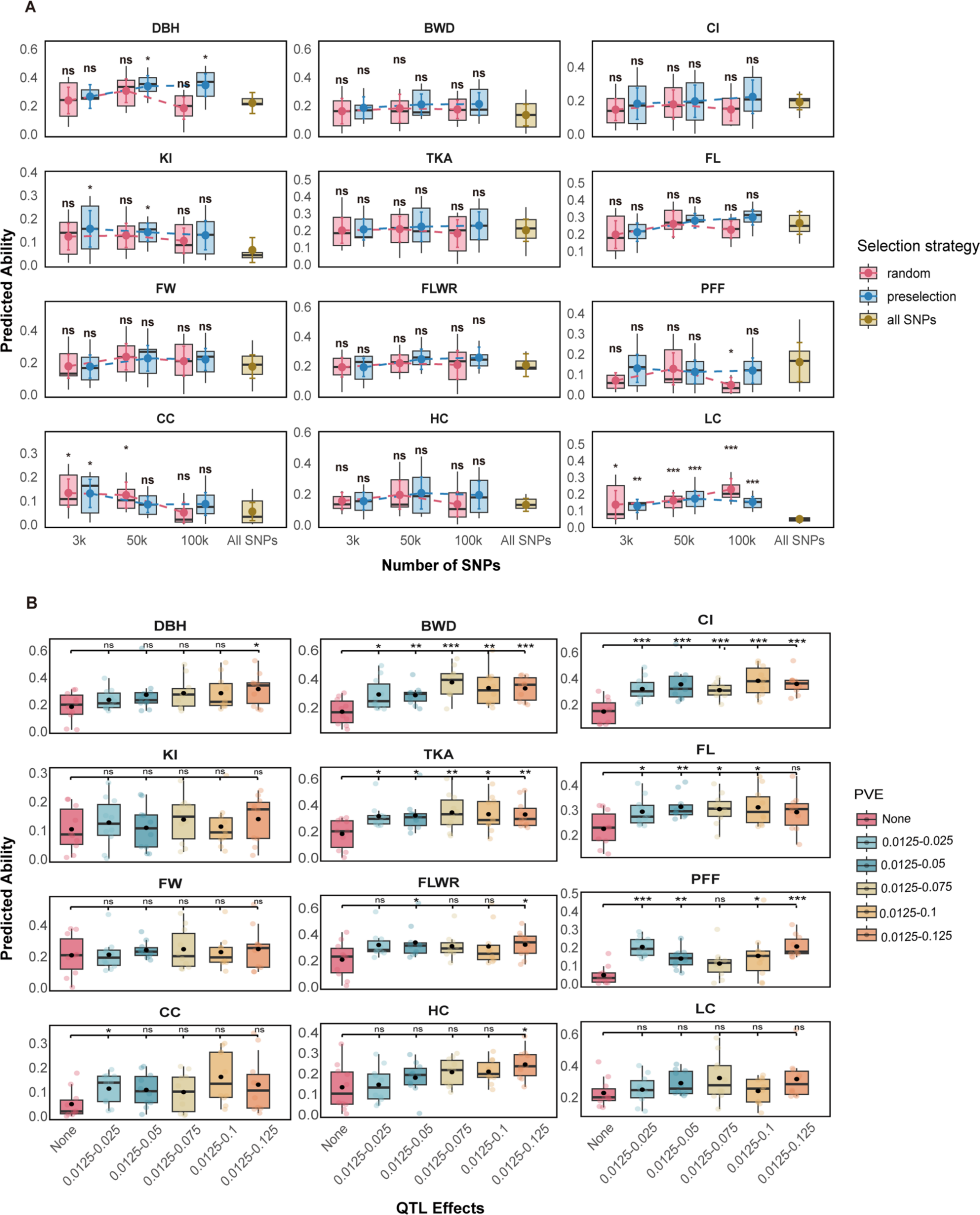
Comparison of PA of Bayesian Lasso models under different SNP selection and fixed-effect strategies. **A** PA across traits using SNPs selected by GWAS-based preselection and random selection. Boxplots show medians and interquartile ranges (whiskers = 1.5 × IQR); faint points are results from tenfold cross-validation; dashed lines trace group means to highlight trends. **B** PA for each trait when SNPs with different phenotypic variance explained (PVE) are included as fixed effects in the model. “None” represents the baseline model without fixed-effect SNPs. Boxplots and points as in panel A; black diamonds indicate group means. Horizontal brackets denote pairwise t-tests versus “None”; ***, **, and * correspond to *p* < 0.001, *p* < 0.01, and *p* < 0.05, respectively, and “ns” indicates not significant

Based on GWAS, we evaluated the influence of two different marker preselection strategy on PA values (Fig. 5). The result showed that PA was improved for only a subset of traits, and none of these improvements reached statistical significance (Fig. 5A, Table S5). For traits such as KI, CC and HC, PA predicted using 3 K or 50 K SNPs was higher than that obtained using 100 K SNPs. However, for most of traits, using 100 K remained the optimal marker density for GS. GS based on the minimum GWAS *P* values expanded the PA range to 0.09—0.35, while random selection of 100 K SNPs resulted in PA values ranging from 0.05 to 0.23, with a maximum improvement of 16.26%.

In contrast, incorporating large effect QTLs as fixed effects in the prediction model led to more pronounced improvements in prediction performance, with PA values ranging from 0.01 to 0.38 and a maximum gain of 23.54%. As the PVE threshold increased from 1.25% to 12.5%, PA exhibited an overall upward trend with some fluctuations. Significant improvements were observed for traits such as BWD, CI, and FL, whereas FW, HC and CC were not statistically significant. Therefore, compared with using significant SNPs as random effect, incorporating QTLs identified based on PVE thresholds as fixed effects resulted in superior prediction performance.

## Discussion

Accurately predicting genetic potential using genomic information and dissecting the genetic architecture of productivity-related traits remain key objectives in forest tree breeding. The present study first evaluated the utility of GS for pulpwood-related traits in slash pine, using a half-sib progeny trial. The observed magnitudes of predictive ability (PA) across 12 complex traits indicate that GS represents a promising tool for accelerating the genetic improvement of quantitative traits in slash pine.

### Performance comparison of statistical models for genomic prediction

Statistical models differ in their capacity to capture LD between markers and QTLs, and to account for genetic relatedness among individuals [44]. Bayesian models often outperform GBLUP and rrBLUP when traits are controlled by a few loci with large effects, as they can shrink small-effect markers toward zero while retaining major-effect loci [45–47]. Conversely, GBLUP assumes equal variance across markers and performs better for highly polygenic traits with evenly distributed small effects (Sahebalam et al., 2025). Despite these theoretical distinctions, prior studies in conifers and other forest trees have reported minimal differences among GBLUP, rrBLUP, and Bayesian models [3, 8, 10], consistent with our findings. In our study, PA positively correlated with trait heritability estimated from GBLUP, BL, and BayesA. However, for wood chemical traits with few markers, GBLUP remained the more accurate model.

### Population structure, LD decay, and marker density

Marker density is a critical determinant of predictive performance [15]. Our results indicated that approximately 100 K SNPs were sufficient to achieve a relatively stable PA close to that obtained with the full marker set for traits such as DBH and BWD in half-sib population. This pattern may be partly attributable to family structure. Compared with full-sib populations, half-sib populations generally contain lower within-family relatedness and greater genetic diversity among individuals and therefore may require higher marker densities to capture LD between markers and QTLs effectively. This interpretation is consistent with previous studies in conifers. In Norway spruce, for example, family structure had a strong influence on marker-density requirements: a full-sib population required only approximately 750 markers to reach comparable prediction accuracy and PA, whereas a half-sib population required approximately 100,000 markers to achieve similar performance; stable accuracies in full-sib populations were generally reached with approximately 4,000–8,000 SNPs [48]. Similarly, a comparison between Douglas-fir full-sib and interior spruce half-sib populations showed higher prediction accuracies in the full-sib population, with accuracy plateauing at approximately 10–15 K SNPs. The authors further emphasized that, at current marker densities, relatedness and family structure contributed more strongly to prediction accuracy than population-wide LD [49]. Comparable patterns have also been reported in black spruce and white spruce [50], where more highly related full-sib populations generally achieved higher GS accuracy with lower marker densities than half-sib populations.

LD decay is an important factor influencing the marker density required for GS because longer LD distance increases the likelihood that markers tag QTLs at a given marker density [17, 51]. In many conifers, LD in natural or weakly structured populations decays within genes or within a few hundred base pairs, suggesting that very high marker densities may be required to directly capture marker–QTL associations [49, 52]. In the present half-sib slash pine population, the LD decay distance at ${r}^{2}=0.2$ was estimated to be 66.29 kb, which is longer than most reported values for conifers [8, 53]. The relatively large LD decay distance observed in our study is likely due to the population structure of the half-sib families, thereby increasing the extent of LD [15, 16, 54]. However, it also highlights the need to consider genetic diversity levels when expanding the population and optimizing marker density [55–57].

Moreover, the GWAS results for multiple wood property traits showed a limited number of significantly associated SNPs (Fig. 2). Only a few traits (e.g., DBH, CI, CC) had significant associations, while others yielded few or no significant signals. This may also reflect the limited resolution due to sparse SNP coverage. Compared to natural populations, the genetic background of breeding populations is relatively narrow, which may also limit the detection power of GWAS. Traditional GWAS is constrained by sample size and statistical methods, and its strict reliance on significance testing results makes it difficult to detect truly existing small-effect loci [58]. Similar GWAS findings have also been reported for biomass traits in *Populus deltoides* [59], growth, wood quality, and disease resistance traits in *Eucalyptus* [60], and growth traits in *Pinus pinaster* [61]. These studies involved both natural and breeding populations, further highlighting the complexity of tree traits.

### Enhancing genomic prediction using GWAS-informed SNPs

The imbalance between a large number of markers and a limited sample size can lead to overfitting and unstable estimation of marker effects in GS models [62]. Previous studies have suggested that integrating GWAS information can mitigate this issue by filtering out redundant or noisy markers, thereby improving prediction accuracy [8]. To ensure the reliability of prediction results, GWAS in this study was performed only within the training population, and the composition of this population varied dynamically across cross-validation folds. This design prevented information leakage between training and validation sets and ensured unbiased evaluation of model performance.

In this study, we first compared the performance of GWAS-guided SNP preselection with that of size-matched random marker subsets. Across traits and marker densities, SNPs preselected according to GWAS *P-values* consistently outperformed random subsets, yielding higher PA. This demonstrates that prioritizing SNPs carrying stronger association signals enhances GS performance by enriching informative loci. Next, we incorporated QTL identified by GWAS into the Bayesian Lasso model as either random or fixed effects. When QTL were modeled as random effects, PA gains were minor and often non-significant, consistent with previous findings in crops [62–64] and forest trees [46]. In contrast, treating large-effect QTL as fixed effects led to significant PA improvements for several growth and wood-quality traits, while chemical traits and fiber width showed weaker responses. This outcome is biologically reasonable: traits influenced by a few moderate-to-large loci benefit from fixed-effect modeling because it minimizes shrinkage of major alleles [65, 66]. The approach is particularly useful for species with large genomes and fast LD decay (e.g., conifers), where random sampling is unlikely to capture informative variants and effect estimates are easily diluted by noise [8, 67].

Similar patterns have been reported in other forest tree species. In Norway spruce, preselecting ~ 100 top GWAS SNPs gave the highest predictive ability for budburst, whereas incorporating major QTL as a fixed effect improved prediction when it explained ≥ 2.5% of phenotypic variance. For more polygenic growth and wood traits, 2000–4000 GWAS-selected SNPs gave similar accuracy to using all markers [8, 48, 68]. Adding multi-trait GWAS QTL as random effects in GS models also increased prediction accuracy by 0.06–0.48 across growth, wood, disease, and leaf traits in Poplar (*Populus deltoides*) [67]. Fixed-effect SNPs substantially boosted predictive ability for fruit traits such as Mango, with large gains in fruit weight and color; combining preselected and fixed-effect SNPs performed best [21].

Overall, GWAS-informed SNP preselection and modeling major QTL can meaningfully improve genomic prediction for traits with simpler, partly oligogenic architectures. For highly polygenic traits, many GWAS-selected SNPs tend to perform similarly to using all markers but still can reduce genotyping costs and model size. These findings have important implications for practical breeding programs. GWAS-informed SNP preselection and incorporation of major QTL can improve prediction efficiency while reducing marker density and computational cost, which is particularly valuable for large-scale genomic selection in conifer breeding populations. In addition, the improved prediction performance for growth and wood-property traits may facilitate earlier and more accurate selection of superior genotypes, thereby accelerating breeding cycles and increasing genetic gain.

## Conclusion

This study demonstrates that genomic selection is a reliable and efficient approach for predicting complex pulpwood-related traits in slash pine. Both GBLUP and Bayesian Lasso provided robust predictive performance, with optimal marker density and model choice varying among traits. Preselecting SNPs based on GWAS *P-value* rankings offered a modest improvement in predictive ability compared with random marker sampling, while incorporating large-effect QTLs as fixed effects produced substantial gains for several wood-quality traits. These results highlight the potential of GWAS-informed strategies to refine genomic prediction accuracy and accelerate breeding efficiency. By integrating association mapping with genomic prediction, this framework provides a practical and scalable route for enhancing pulpwood yield and quality in conifer breeding programs.

## Supplementary Information

Supplementary Material 1.

## Data Availability

The original liquid-phase probe capture reads and SNP data during the current study are available in the China National GeneBank DataBase (CNGBdb) under project accession number CNP0009094 (https://db.cngb.org/data_resources/project/CNP0009094/). All data generated or analyzed during this study are included in this published article and its supplementary information files.

## Abbreviations

GWAS: Genome-wide association study
GS: Genome selection
QTL: Quantitative Trait loci
FL: Fiber length
FW: Fiber width
KI: Kink index
TKA: Total kink angle
CI: Curl index
PFF: Percentage of fines
GBLUP: Genomic best linear unbiased prediction
BRR: Bayesian Ridge Regression
BL: Bayesian LASSO
TP: Training population
PA: Predictive ability
PVE: Proportion of variance explained
VP: Validation population
GEBVs: Genomic estimated breeding values
PC: Prediction accuracy
LC: Content of lignin
CC: Cellulose content
HC: Hemicellulose content
AIC: Akaike information criterion
BIC: Bayesian information criterion
LD: Linkage disequilibrium
FDR: False discovery rate
BWD: Basic wood density
DBH: Diameter at breast height
